# Immunogenicity after outbreak response immunization activities among young healthcare workers with secondary vaccine failure during the measles epidemic in Korea, 2019

**DOI:** 10.1186/s12879-022-07511-2

**Published:** 2022-06-08

**Authors:** Hyeri Seok, Erica Españo, Jooyun Kim, Ji Hoon Jeon, Won Suk Choi, Yun-Kyung Kim, Jeong-Ki Kim, Dae Won Park

**Affiliations:** 1 Division of Infectious Diseases, Department of Medicine, Korea University Ansan Hospital, Korea University College of Medicine, 123 Jeokgeum-ro, Danwon-gu, 15355 Ansan, Republic of Korea; 2 Department of Pharmacy, Korea University College of Pharmacy, 2511 Sejong-ro, Sejong, 30019 Republic of Korea; 3 Department of Pediatrics, Korea University Ansan Hospital, Korea University College of Medicine, 123 Jeokgeum-ro, Danwon-gu, 15355, Ansan, Republic of Korea

**Keywords:** Measles, Measles-Mumps-Rubella Vaccine, Vaccine immunogenicity, Antibody affinity, Neutralization tests

## Abstract

**Background:**

Despite high vaccination coverage, measles outbreaks have been reported in measles elimination countries, especially among healthcare workers in their 20 and 30 s. This study was designed to identify measles-susceptible individuals and to evaluate whether primary or secondary vaccine failure occurred during measles outbreak response immunization (ORI) activities.

**Methods:**

The study population was divided into three groups as follows: natural immunity group (Group 1), vaccine-induced immunity group (Group 2), and vaccine failure group (Group 3). We evaluated the immunogenicity of measles among healthcare workers using three methods—enzyme-linked immunoassays, plaque reduction neutralization tests, and avidity assays. The results were assessed at baseline, 4 weeks after, and 6 months after the completion of measles-mumps-rubella (MMR) vaccination.

**Results:**

In total, 120 subjects were enrolled, with 40 subjects in each group. The median age of Group 3 was 29 years, which was significantly lower than that of the other groups. The baseline negative measles virus (MeV) IgG in Group 3 increased to a median value of 165 AU/mL at 4 weeks after ORI and was lower than that in Groups 1 and 2. The median neutralizing antibody titer was highest in Group 1, and this was significantly different from that in Group 2 or Group 3 at 4 weeks (944 vs. 405 vs. 482 mIU/mL, *P* = 0.001) and 6 months (826 vs. 401 vs. 470, *P* = 0.011) after ORI. The rates of high MeV avidity IgG were highest in Group 2, and these were significantly different from those in Groups 1 or 3 at 4 weeks (77.5 vs. 90% vs. 88.6%, *P* = 0.03) and 6 months (81 vs. 94.8 vs. 82.1%, *P* = 0.01) after ORI.

**Conclusions:**

Considering the MeV-neutralizing antibodies and IgG avidity after MMR vaccination in measles-susceptible group, vaccine failure is inferred as secondary vaccine failure, and further data regarding the maintenance of immunogenicity are needed based on long-term data. The MeV-neutralizing antibody levels were highest in the natural immunity group, and the primary vaccine-induced immunity group showed the highest rates of high MeV IgG avidity.

## Introduction

Measles is a highly contagious and vaccine-preventable disease that has not been completely eradicated yet. The measles virus (MeV) was first discovered in 1757. Before the first measles vaccine was developed in 1963, hundreds of thousands of children worldwide were diagnosed with measles, and thousands of children died each year. Through the worldwide advancement of measles vaccine development, two doses of the measles-containing vaccine (MCV), the first dose at 9 months or 12–15 months of age and the second at 15–18 months or 4–6 years of age, are used in most countries, although there are differences in the timing and catch-up of the second dose in each country. MCV coverage is reported to be more than 90% in developing countries, and measles elimination has been verified by the World Health Organization (WHO) in the Americas, Oceania, East Asia, and parts of Europe [1]. Despite high vaccination rates, measles has not been eradicated, and the disease accounts for more than 140,000 deaths in children under the age of 5 years worldwide [2]. Cases or outbreaks of measles are reported in countries that have eliminated it, and most cases involve unvaccinated individuals owing to cultural reasons or introduction from measles-endemic countries. In 2019, the United States had the highest number of cases (1282) since 1992, and Europe reported a total of 82,596 cases and 72 deaths in 2018 [3, 4].

In Korea, measles caused serious deaths in the pediatric population before the introduction of measles vaccination, and about 1 million children, or about 20% of the children’s population, were infected with measles, resulting in 20,000 deaths [5]. The national free vaccination program was introduced in 1985, and the requirement for compulsory second doses of vaccination before elementary school entry was established in 1997. In 2006, Korea complied with all standards of measles elimination presented by the WHO, and measles elimination was verified by the WHO in 2014 [6]. However, Korea also reported the highest number of measles cases (194) in 2019 [7]. With regard to the route of the infection, 86 individuals (44.3%) had a history of overseas travel and 74 (38.1%) had nosocomial exposure in hospitals. One hundred twenty patients (61.9%) were in the 20–39 years age group. The characteristic features of this epidemic were measles infections in young healthcare workers (HCWs) after exposure to measles patients and breakthrough measles infections among previously immunized people with two-dose measles vaccination [8–10]. Under the Occupational Safety and Health Act, medical institutions were encouraged to determine the measles immunity status of HCWs and implement outbreak response immunization (ORI) activities with two doses of MCV for susceptible workers [7].

Breakthrough infections among previously immunized people are a common problem in countries with high vaccination coverage; moreover, measles elimination has been sustained over several decades and appears to occur when vaccinated groups are not exposed to measles, which might induce natural boosting [11–14]. While implementing ORI activities in highly vaccinated populations with low measles incidences, targeted campaigns could be of greater benefit [15, 16] and differentiating primary or secondary vaccine failure is important for strategies to control measles in settings in which the disease has been eliminated [17]. The aim of this study was to investigate the changes in and correlations between neutralizing antibody titers and avidity over time after ORI among three populations, specifically those with natural infection, those seropositive after being vaccinated with two doses of the measles-mumps-rubella (MMR) vaccine, and those who were seronegative after two doses of the vaccine. Another objective was to determine whether primary or secondary vaccine failure occurred in seronegative populations.

## Subjects and methods

### ORI activities

In February 2019, the Korea University Ansan Hospital conducted MeV IgG tests on all HCWs in accordance with an administrative order during measles outbreaks in Ansan, Gyeonggi-do. Among all 1278 HCWs, there was one confirmed case of community-transmitted measles. 1202 (94.1%) were positive for IgG, 21 (1.6%) were equivocal, and 55 (4.3%) were negative. All seronegative or equivocal workers received two doses of the MMR vaccine 4 weeks apart. The vaccine used was manufactured by Merck Sharp and Dohme (USA) and contained the Enders’ Edmonston measles strain (strength > 3.0 log tissue culture infectious doses [TCID50]), Jeryl Lynn mumps strain (> 4.1 log TCID50), and Wistar RA 27/3 rubella strain (> 3.0 log TCID50).

### Study design and population

The study population was divided into three groups of 40 individuals each, namely, the natural immunity group (Group 1), the vaccine-induced immunity group (Group 2), and vaccine failure group (Group 3). The criteria for each group were as follows: Group 1 was classified as MeV-IgG seropositive subjects with a history of infection or born before 1968. Group 2 was classified as MeV IgG-seropositive people with primary measles vaccination without a history of infection and born after 1968. Group 3 was classified as MeV IgG-seronegative subjects with primary measles vaccination. In Korea, the measles vaccine was first introduced in 1965, and the seroprevalence rate was 95.4% in the 30–34 year-old group in the national measles immunogenicity study in 2002. Therefore, those born before 1968 were considered to have natural immunity to measles through expert consensus. Plasma was obtained from all groups at baseline and 4 and 6 months after ORI; two doses of the MMR vaccine were administered to seronegative subjects. This study was conducted on those who agreed to participate in this study among all healthcare workers who were tested for measles. Based on 40 of the 55 seronegative subjects who agreed to participate in the study, 80 seropositive subjects who agreed to participate in the study were included.

### MeV IgG enzyme-linked immunoassay (ELISA)

ELISA is an easy, rapid, and automated method that is widely used for measuring antibodies. Since ELISA measures the total measles-specific antibody titer, its specificity is lower than that of other tests. The LIAISON Measles IgG ELISA kit (DiaSorin, USA) was used to measure measles IgG, and the experiment was performed as per the protocol. The results, specifically < 13.5 AU/mL, 13.5–16.4 AU/mL, and > 16.5 AU/mL, were interpreted as negative, equivocal, and positive, respectively.

### Plaque reduction neutralization test (PRNT)

PRNT, which detects functional neutralizing antibodies, is regarded as the gold standard method for assessing measles immunity [18]. PRNT is more sensitive than ELISA in that it detects antibodies against nucleocapsid proteins, whereas PRNTs detect neutralizing antibodies against hemagglutinin and fusion proteins [19]. PRNT was performed with Vero cells infected with a low-passage Edmonston strain of MeV, based on a previous method [18, 20]. The interpretation of the results was divided into four categories according to previous studies as follows: negative (< 8 mIU/mL), low MeV-neutralizing antibody level (8–120 mIU/mL), medium MeV-neutralizing antibody level (121–900 mIU/mL), and high MeV-neutralizing antibody level (> 900 mIU/mL). A MeV neutralizing antibody level > 120 mIU/mL was interpreted as immune to measles, with < 120 mIU/mL considered susceptible to measles.

### MeV IgG antibody avidity

An MeV IgG avidity assay can differentiate primary or secondary failure in that virus-specific high-avidity antibodies are associated with pre-existing memory B cells [21]. However, cases of reinfection with a history of vaccination or measles might be detected as high avidity IgG [22]. The measles virus IgG ELISA kit (avidity; Abcam, Cambridge, UK) was used to measure measles-specific IgG avidity, and the experiment was performed as per the manufacturer’s protocol. The results, specifically < 45%, 45–55%, and > 55%, were interpreted as low, equivocal, and high IgG avidity, respectively. Low IgG avidity can be interpreted as a primary infection acquired within the past 2 months.

### Statistical analyses

For comparisons, the Pearson χ² and Fisher’s exact tests were used for categorical variables, and Student’s t-tests and Mann–Whitney U tests were used for continuous variables, as appropriate. Multivariate logistic regression was used to evaluate the association among the ELISA, PRNT, and avidity assays. A reverse cumulative distribution curve was used to compare shifts between variables over time. All statistical tests were two-tailed, and *P*-values ≤ 0.05 were considered statistically significant. All statistical analyses were performed using SPSS Statistics version 20.0 for Windows (IBM Corp., Armonk, NY, USA).

## Results

**Fig. 1:**
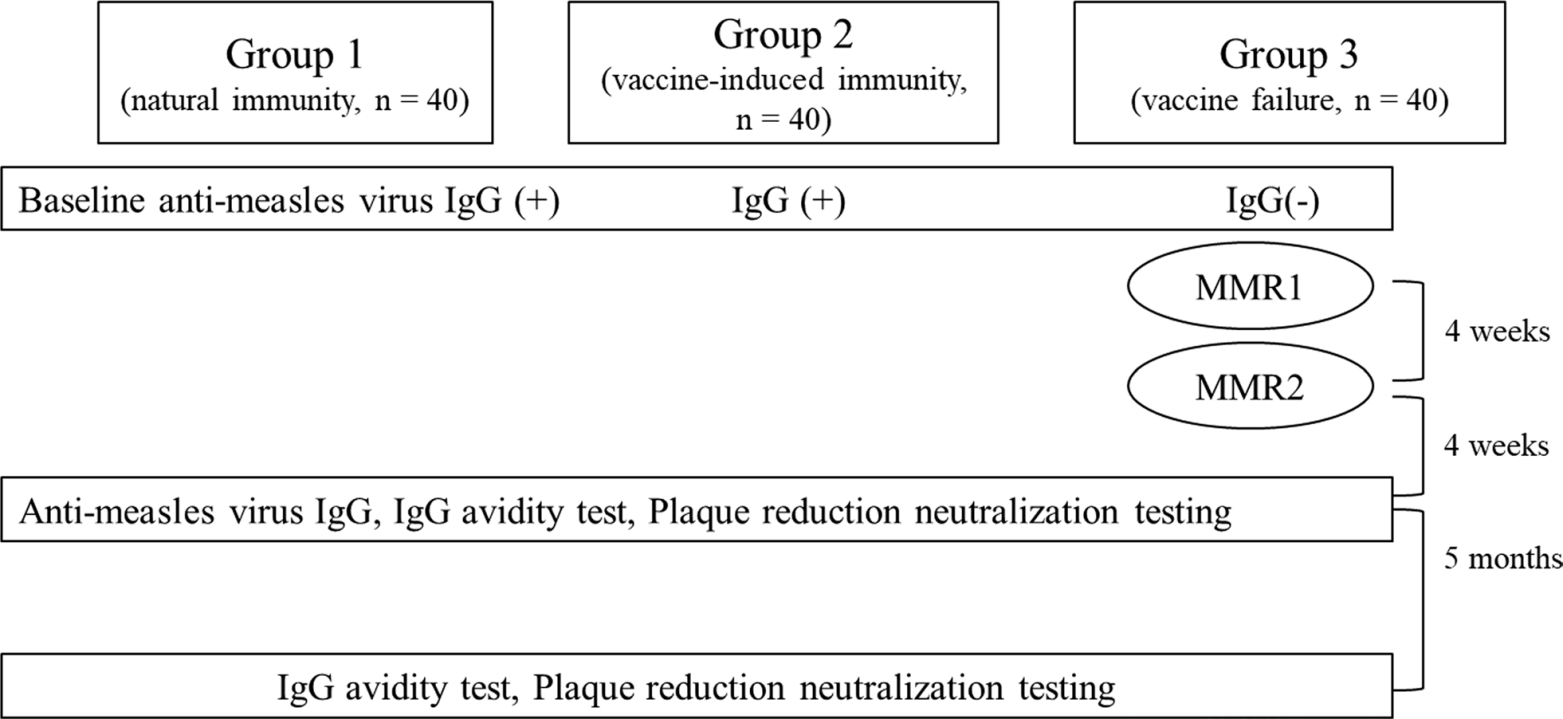
Flowchart of population in this study. *MMR* measles-mumps-rubella

**Fig. 2:**
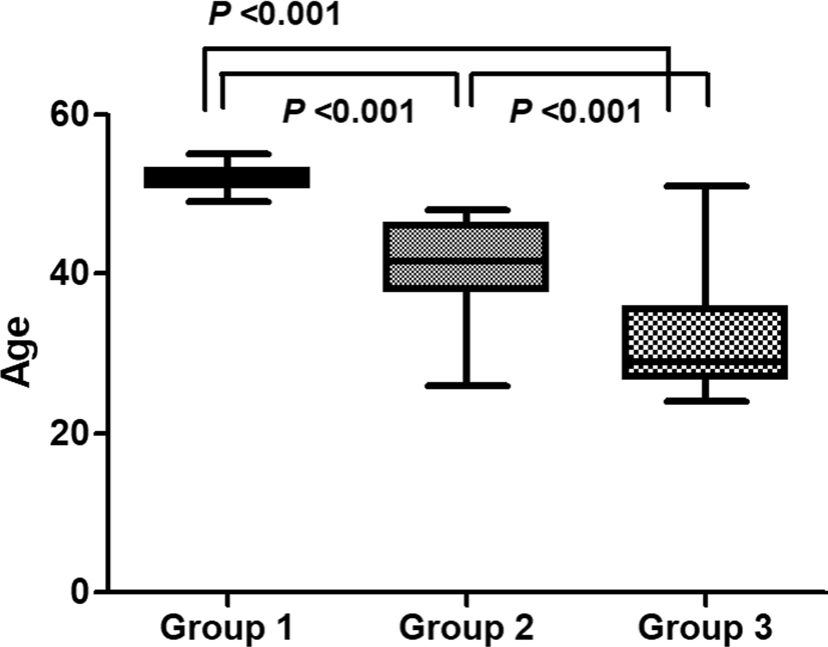
Comparison of age of each group according to measles immunity status. Group 1: natural immunity; Group 2: vaccine-induced immunity; Group 3: vaccine failure

In total, 120 subjects participated in the study, and each of the three groups consisted of 40 subjects (Fig. 1). Baseline characteristics of each group are presented in Table 1. The average ages of the natural immunity, vaccine-induced immunity, and vaccine failure groups were 52, 41, and 32 years, respectively, which were significantly different (Fig. 2). Groups 1 and 2 were seropositive for baseline anti-measles IgG and Group 3 was seronegative, and there was no difference in the median values of anti-measles IgG between Groups 1 and 2. The significantly lower baseline measles IgG level in Group 3 than that in the other groups increased at 1 month after ORI (7 vs. 165 AU/mL). When the baseline anti-measles IgG levels among Groups 1, 2, and Group 3 at 1 month after ORI were compared, there was no significant difference.

**Table 1:** Baseline characteristics and comparison of immunogenicity in each group according to measles immunity status

	Group 1 (natural immunity)	Group 2 (vaccine-induced immunity)	Group 3 (vaccine failure)	*P* value
Age	52 ± 1 (51–53)	41 ± 5 (38–46)	32 ± 6 (27–35)	< 0.001
Male	15 (37.5)	5 (12.5)	11 (27.5)	0.038
Baseline				
Anti-measles IgG, AU/mL	300 (201–300)	283 (130–300)	7 (0.6–11)	< 0.001
1 month after MMR vaccination				
Anti-measles IgG, AU/mL	NA	NA	165 (83–300)	0.047*
Neutralizing Ab, mIU/mL	944 (482–1249)	405 (301–704)	482 (272–780)	0.001
Above medium (> 121)	39/40 (97.5)	38/40 (95)	40/40 (100)	1.00
High (> 900)	23/40 (57.5)	7/40 (17.5)	7/40 (7.5)	< 0.001
Avidity (%)	63 (58–68)	70 (67–74)	68 (61–73)	0.003
High avidity (55%)	31/40 (77.5)	36/40 (90)	31/35 (88.6)	0.030
6 months after MMR vaccination				
Neutralizing Ab, mIU/mL	826 (441–1279)	401 (287–653)	470 (280–806)	0.011
Above medium (> 121)	36/37 (97.3)	36/40 (90)	28/28 (100)	0.035
High (> 900)	17/37 (45.9)	6/40 (15)	5/28 (17.9)	< 0.001
Avidity (%)	65 (57–75)	71 (66–75)	61 (58–69)	0.005
High avidity (55%)	30/37 (81)	36/38 (94.8)	23/28 (82.1)	0.010

*Comparison of baseline anti-measles IgG values of groups 1 and 2 and anti-measles IgG at 1 month after MMR vaccination in group 3; MMR: measles-mumps-rubella; NA: not applicable

We compared MeV-neutralizing antibodies in each group at 1 and 6 months after ORI. The median values of MeV-neutralizing antibodies at 1 month after ORI were 944, 405, and 482 mIU/mL in Groups 1, 2, and 3, respectively, and these were significantly higher in Group 1. The above medium- or high-MeV neutralizing antibody levels at 1 month after ORI accounted for 95% of those in all three groups, and these showed a protective effect. The rates of high MeV-neutralizing antibody levels at 1 month after ORI were 57.5%, 17.5%, and 7.5% in Groups 1, 2, and 3, respectively, and these were significantly higher in Group 1. This trend remained similar to that observed at 6 months after ORI. The median values of neutralizing antibodies at 6 months after ORI were 826, 401, and 470 mIU/mL in Groups 1, 2, and 3, respectively, and these values were significantly higher in Group 1. Above medium- or high-neutralizing antibody levels at 6 months after ORI accounted for 97% of those in all three groups. The rates of high levels of MeV neutralizing antibodies at 6 months after ORI were 45.9%, 15%, and 17.9% in Groups 1, 2, and 3, respectively, and these were significantly higher in Group 1. In the avidity assay, the rates of high avidity in the three groups were 77% or more at 1 month after MMR2 and 81% or more at 6 months after MMR2. In the comparison of avidity assays among groups, the median level of IgG avidity was the highest in Group 2 at 1 month after MMR2, and this was maintained at 6 months.

### MeV-neutralizing antibody concentrations in Group 3 (vaccine failure group)

**Fig. 3:**
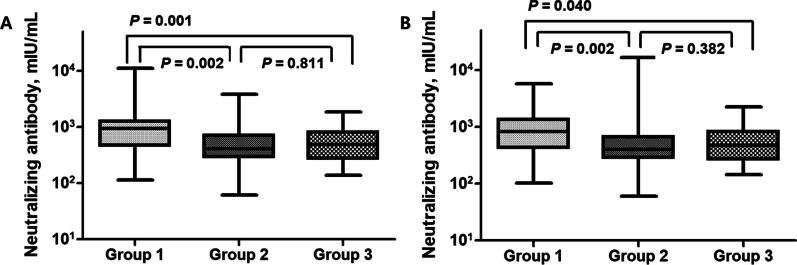
Measles virus (MeV)-neutralizing antibody concentrations in each group according to measles immunity status. **A** MeV-neutralizing antibody concentrations 1 month after MMR2. **B** MeV-neutralizing antibody concentrations 6 months after MMR2. Group 1: natural immunity; Group 2: immune-induced immunity; Group 3: vaccine failure, MMR: measles-mumps-rubella

**Fig. 4:**
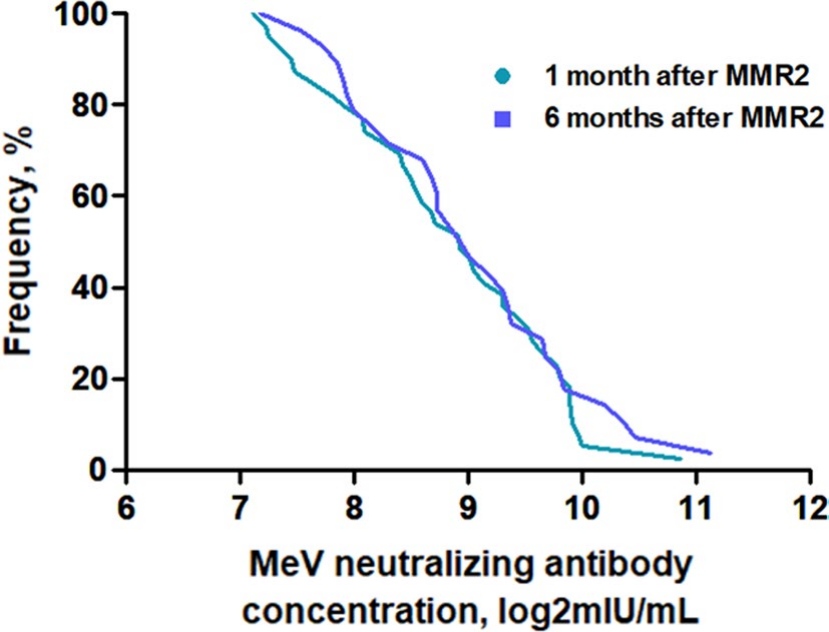
Reverse cumulative distribution curve of measles virus (MeV) antibody concentrations at 1 month and 6 months after MMR2

MeV-neutralizing antibody concentrations over time in Group 3 were analyzed (Fig. 3). MeV-neutralizing antibody levels in Group 3 at 1 month after ORI did not show a significant difference from those of Group 2 and were lower than those of Group (1) MeV-neutralizing antibody levels at 6 months also showed no difference compared with those in Group (2) MeV-neutralizing antibody concentrations, which were elevated at 1 month after ORI in Group 3, were maintained at 6 months, without a significant difference between the two groups (*P* = 0.691). Logistic regression analysis revealed that neutralizing antibody concentrations at 1 and 6 months after ORI showed a statistically significant correlation (*P* < 0.001). The rates of high neutralizing antibody levels at 1 and 6 months after ORI showed no differences (*P* = 0.626). The reverse cumulative distribution curve also showed that there was almost no shift in the MeV antibody concentration at 1 and 6 months after ORI (Fig. 4). MeV-neutralizing antibody titers showed no statistical relationship with anti-measles virus IgG levels at 1 and 6 months after ORI.

### MeV IgG antibody avidity in Group 3 (vaccine failure group)

**Fig. 5:**
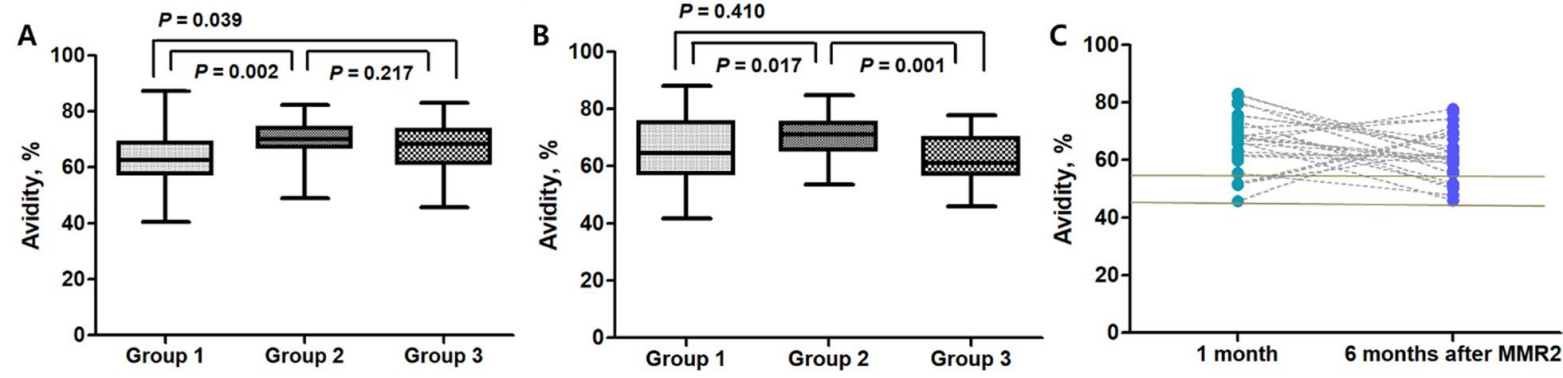
Measles virus (MeV) IgG antibody avidity in each group according to measles immunity status. **A** MeV IgG antibody avidity at 1 month after MMR2. **B** MeV IgG antibody avidity at 6 months after MMR2. **C** Individual time-dependent MeV IgG avidity at 1 and 6 months after MMR2. Group 1: natural immunity; Group 2: vaccine-induced immunity; Group 3: vaccine failure, MMR: measles-mumps-rubella

MeV IgG antibody avidity over time in Group 3 was analyzed (Fig. 5). MeV IgG antibody avidity in Group 3 at 1 month after ORI was significantly higher than that in Group 1, and there was no significant difference from that in Group 2. MeV IgG antibody avidity in Group 3 at 6 months after ORI was significantly lower than that in Group 2 and was not different from that in Group 1. Logistic regression analysis revealed that MeV IgG avidity at 1 and 6 months after ORI showed no statistically significant association (*P* = 0.213). MeV IgG avidity, which was elevated at 1 month after ORI in Group 3, was maintained at 6 months, but MeV IgG avidity at 1 month was higher than that at 6 months (*P* = 0.049). Individual time-dependent MeV IgG avidity did not show consistently high or low values, and there was no low IgG avidity at 1 and 6 months after ORI. MeV IgG avidity showed no statistical relationship with anti-measles virus IgG levels at 1 and 6 months after ORI.

## Discussion

To the best of our knowledge, this is the first study to evaluate the immunogenicity of measles-susceptible individuals using various methods to differentiate between primary or secondary vaccine failure. MCV failure in Korea can be due to secondary vaccine failure based on the results that both MeV IgG avidity and neutralizing antibody concentrations in measles-susceptible individuals increased 1 month after ORI. Considering that Korea has maintained the requirements for measles elimination for years, secondary vaccine failure might be a similar phenomenon in other measles-eliminated countries [23–26].

Age was significantly different among groups that were classified according to measles immunity acquisition; older age was associated with higher natural immunity rates, whereas younger age was associated with more individuals in the non-responder or unvaccinated groups. This is consistent with previous studies showing that before the measles elimination period, the elderly maintained measles immunity through natural infection and boosting, and as age decreases, individuals have maintained measles immunity through vaccine-induced immunity and natural boosting [27, 28]. Individuals born after measles elimination maintained immunity against the disease only through vaccine-induced immunity, without the opportunity for natural boosting. In part, the age difference may be caused by the criteria defining the group in this study, which is an inevitable confounding factor considering the history of measles.

MeV-neutralizing antibody levels were higher in the natural immunity group than in the vaccine immunity group, regardless of the timing of vaccination. MeV-neutralizing antibody levels in the natural immunity group had a higher median value, and the proportion of those with a high level was also significantly higher than that in the other groups. This is consistent with previous studies and is probably because the starting antibody titer in the naturally immune group was higher than that in the group with vaccine immunity [29, 30]. In Group 3, there was no difference in the median MeV-neutralizing antibody levels at 1 month and 6 months after MMR vaccination, which might be because protective efficacy against measles is acquired at 1 month after vaccination. The ratio of high levels of neutralizing antibodies showed a tendency to increase 6 months after MMR vaccination. This result is different from that of previous studies in that the MeV-neutralizing antibody level showed a decreasing trend over time after vaccination, and the evaluation time was previously from 1 year after vaccination [30, 31].

As the measles susceptible group, represented by a young age, has received two doses of the MMR vaccination before entering elementary school since 1997, seronegative individuals for measles could be interpreted as non-responders given the vaccination history. However, not every immunization certificate can be assured because the national immunization program was developed later. Vaccine failure in the measles-susceptible group is considered secondary failure if the vaccination record is clear. However, determining the booster dose is another question because MeV IgG does not correlate with neutralizing antibody concentrations. Previous studies have suggested that a third dose of the MMR vaccine might be necessary, but this is for mumps outbreaks not measles [32–34]. During small outbreaks of measles in Korea in 2019, breakthrough measles infections in HCWs with a clear vaccination history occurred, and most cases were asymptomatic or atypical infections with mild symptoms, which did not induce outbreaks owing to low infectivity [13]. However, breakthrough infections in all individuals with a clear MCV vaccination history cannot guarantee atypical infection or low infectivity, and the probability of measles infections in HCWs is relatively high considering the Ro value. Based on this study, re-evaluation of the immunogenicity against measles and the administration of a booster dose should be considered in high-risk groups, including HCWs in measles-susceptible individuals.

Whether there is a correlation between neutralizing antibody concentrations and antibody binding strength has been controversial in previous studies [31, 35]. In our study, MeV IgG avidity was highest in Group 2 (vaccine-induced immunity and natural boosting), which was similar to the results of a previous study [36], and the hypotheses we considered are as follows. First, antibody avidity is the total noncovalent interaction between antigens and antibodies through somatic hypermutations in MeV-specific B cells. However, the increase in overall affinity does not mean that the specific IgG levels for H and F proteins, which are related to neutralizing capacity, are high [37]. In the process of immune maturation after MCV vaccination, some hypermutations other than those related to specific epitopes involved in neutralization might have contributed to high avidity. Second, the high avidity in the vaccinated group could be related to the specific IgG isotype. A previous study showed that the IgG isotype was different from that with natural infection and after vaccination, with a significant decrease in IgG4 in the post-vaccination group [38]. Another study showed that IgG2 levels significantly increased during the chronic covalent period in the natural infection group [39]. An increase in the relative IgG1 ratio in the vaccinated group might be associated with high avidity, and further studies are needed because of the limited number of studies on isotypes in the vaccinated or natural infection group. Finally, it is necessary to determine the accuracy of the IgG avidity test. A previous review suggested that the standardization of modified ELISAs for antibody avidity tests is needed based on different results when different test methods are used [40]. Thus, the results might have been due to errors in non-standardized testing methods.

The strength of this study is that it classified groups according to immunity status against measles and simultaneously compared immunogenicity with various methods. In previous studies, to evaluate the cause of measles vaccine failure, a neutralizing antibody or antibody avidity assay was performed, but there was no study that conducted both tests. The limitation of this study is the possibility of selection bias because it was performed based on HCWs from a single institution. However, the findings could be helpful in establishing a plan to respond to measles outbreaks in medical institutions in the future by studying HCWs who are at a high risk of measles transmission. Another limitation of this study is that the data account for only up to 6 months after the vaccination of measles-susceptible individuals, and further long-term seroprevalence data are required. Finally, there may be limitations related to the reliability and possible errors of the tests for the measles immunogenicity in this study.

In conclusion, measles vaccine failure in measles-susceptible group in Korea that sustain measles elimination may be due to secondary vaccine failure based on this study. This suggests that a re-evaluation of measles immunogenicity and booster doses for MCV should be considered in such populations, and especially in increased risk for exposure to measles such as HCWs.

## Data Availability

All data used in analysis of this manuscript is freely available by contacting the corresponding author.

## Abbreviations

ELISA: Enzyme-linked immunoassay
HCW: Healthcare worker
MCV: Measles-containing vaccine
MeV: Measles virus
MMR: Measles-mumps-rubella
ORI: Outbreak response immunization
PRNT: Plaque reduction neutralization test
TCID: Tissue culture infectious doses
WHO: World Health Organization
